# Development of clinical trials for non-small cell lung cancer drugs in China from 2005 to 2023

**DOI:** 10.3389/fmed.2023.1239351

**Published:** 2023-11-16

**Authors:** Wanying Jia, Haiyan Yu, Li Song, Jian Wang, Shuyu Niu, Guojie Zang, Mingjie Liang, Jinwei Liu, Risu Na

**Affiliations:** ^1^Department of Pharmacy, Chi Feng Municipal Hospital, Chifeng, China; ^2^Qingdao Women and Children’s Hospital, National Drug Clinical Trial Institute Office, Qingdao, China; ^3^Department of Pharmacy Supplement, Chi Feng Municipal Hospital, Chifeng, China; ^4^Chifeng Clinical Medicine College of Inner Mongolia Medical University, Chifeng, China; ^5^Clinical Science of Stomatology, Chi Feng Municipal Hospital, Chifeng, China

**Keywords:** non-small cell lung cancer, China, drug clinical trials, registration application, statistical analysis

## Abstract

**Objective:**

Over the past few decades, the development of anti-cancer drugs in China has made outstanding achievements based on the support of national policies. To assess the progress of non-small cell lung cancer (NSCLC) drugs, we conducted a statistical analysis of clinical trials of drugs targeting NSCLC in China from 2005 to 2023.

**Methods:**

We downloaded, screened and analysed the data from three official websites, the Centre for Drug Evaluation of China National Medical Products Administration website (NMPA), ClinicalTrials.gov and the Chinese Clinical Trial Registry (ChiCTR).

**Results:**

From January 1, 2005 to April 15, 2023, a total of 1,357 drug clinical trials that met the standards were included, and the number of registered drug clinical trials has been increasing year by year, reaching the maximum of 199 in 2021. Among them, the maximum of 462 items (34.05%) in phase II clinical trials, followed by 333 (24.54%) in phase III clinical trials, and 139 (10.24%) in phase IV clinical trials. In all drug clinical trials, industry sponsored trials (ISTs) have 722 items (53.21%), which are higher than investigator-initiated trials (IITs). The clinical trials of chemical drugs have a maximum of 723 items (53.28%), while biopharmaceuticals have grown rapidly in the past 10 years, with a total of 374 (27.56%), and 48.19% of the drug clinical trials of combined medication. In addition, the geographical distribution of the leading units and participating units of Chinese drug clinical trials are uneven, and economic regions such as Beijing, Shanghai, Jiangsu are obviously ahead of other regions.

**Conclusion:**

From 2005 to 2023, the clinical trials of registered drugs for the treatment of NSCLC increased rapidly. Among them, due to the development of immunotherapy, the clinical trials of biopharmaceuticals and drugs for combined medication are growing most rapidly, while the exploration of the original drugs is a little far from enough. Our research provides a direction for the future drug clinical trials of NSCLC, laying foundation for further extending the survival rate of patients with NSCLC.

## 1 Introduction

Lung cancer is the leading cause of cancer-related deaths worldwide. Non-small cell lung cancer (NSCLC) accounts for 85% of lung cancers, and the 5 years overall survival (OS) rate is around 15% (1, 2). There are different subtypes of NSCLC, classified as squamous cell carcinoma (SqNSCLC), adenocarcinoma, large cell carcinoma and more poorly differentiated variants (3). According to the China National Cancer Centre report, China had 4.064 million new cases of malignant tumours and 2.414 million deaths in 2016, including 828,000 new cases of lung cancer and 657,000 deaths (4).

Clinical trials are the gold standard for the evaluation of the safety and efficacy of therapeutics and the generation of evidence-based knowledge in the field of medicine (5, 6). The legitimacy of clinical trials can be ensured by informed consent, data collection of clinical trial processes, and quality assurance systems. China launched drug regulatory reforms in 2015, which include a 60 days standard approval system for clinical trial applications, expedited program (such as conditional approval), acceptance of overseas clinical trial data and the introduction of patent term extension, which have greatly facilitated the development of new drugs (7, 8). The standardized good clinical practice (GCP) standards in China enabled the scientific and reliable test results of drug clinical trials, and also protect the rights and interests of the subjects.

Surgical resection of lung tumors is the preferred therapeutic approach when early-stage disease is detected. Unfortunately, most patients present with advanced, inoperable disease because lung cancer is largely asymptomatic in its early stages (9). Therefore, drug treatment has become the best way to relieve symptoms. The advent of genetic and molecular technologies has been accompanied by dramatic changes in the treatment options for NSCLC. In particular, a large number of genomic alterations in genes that regulate cell proliferation and differentiation have been identified as driver mutations for lung cancer treatment (10–12). Several treatment modalities are being used including surgery, radiation therapy, chemotherapy, targeted therapy, laser therapy, photodynamic therapy, radiofrequency ablation, cryosurgery, electrocautery, and watchful waiting. New modalities such as immunotherapy, combination therapies and chemoprevention are being tested in clinical trials (3, 13). In immunotherapy, immune checkpoint inhibitors may be able to prolong the survival cycle and improve the prognosis of patients to some extent by modulating the immune response involving T cells to clear the tumour cells in the patient (14, 15). Various drug therapies for NSCLC are flourishing, but to date, no analytical overview of drug clinical trial for NSCLC has been undertaken.

In this study, we present an analysis of clinical trials of drugs for non-small cell lung cancer conducted in China from 2005–2023. Our study provides direction to drug trial applicants and research institutions, and provides a basis for the use of drugs and treatment plan for medical institutions.

## 2 Materials and methods

### 2.1 Data sources

Our drug clinical trial data is obtained from three official websites, namely the Centre for Drug Evaluation of China National Medical Products Administration website (NMPA),^1^ ClinicalTrials.gov^2^, and the Chinese Clinical Trial Registry (ChiCTR)^3^ (16). The Chinese Clinical Trial Registry was first established in 2005. Therefore, January 1, 2005 was chosen as the start date and April 15, 2023 was chosen as the cut-off date for this study. Any clinical trials of drugs outside this period were excluded.

### 2.2 Search strategy and selection criteria

A total of 537,178 drug clinical trials were retrieved from the three websites. In NMPA and ChiCRT, our search terms were non-small cell lung cancer, and we searched for 679 and 740 studies, respectively, in the above-mentioned websites. In ClinicalTrial.gov, we entered the keyword non-small cell lung cancer in “condition or disease” and also entered China in “country” to obtain 1,255 studies.

The inclusion criteria in this study were: (1) the drug clinical trial had to be for non-small cell lung cancer; (2) the study site was within China; (3) the trial was for drug treatment related to NSCLC; (4) one of the evaluation indicators had to be the efficacy of the drug treatment; and (5) the study was started between January 1, 2005 and April 15, 2023. Exclusion criteria were as follows: (1) studies started before 1 January 2005 and after 15 April 2023; (2) some clinical trials focused on predictive as well as prognostic markers for NSCLC; (3) inclusion criteria for drug clinical trials covering patients with solid tumours other than NSCLC. Chemotherapy alone as well as cellular therapies were not included in the study during the trial selection process.

**Figure 1:**
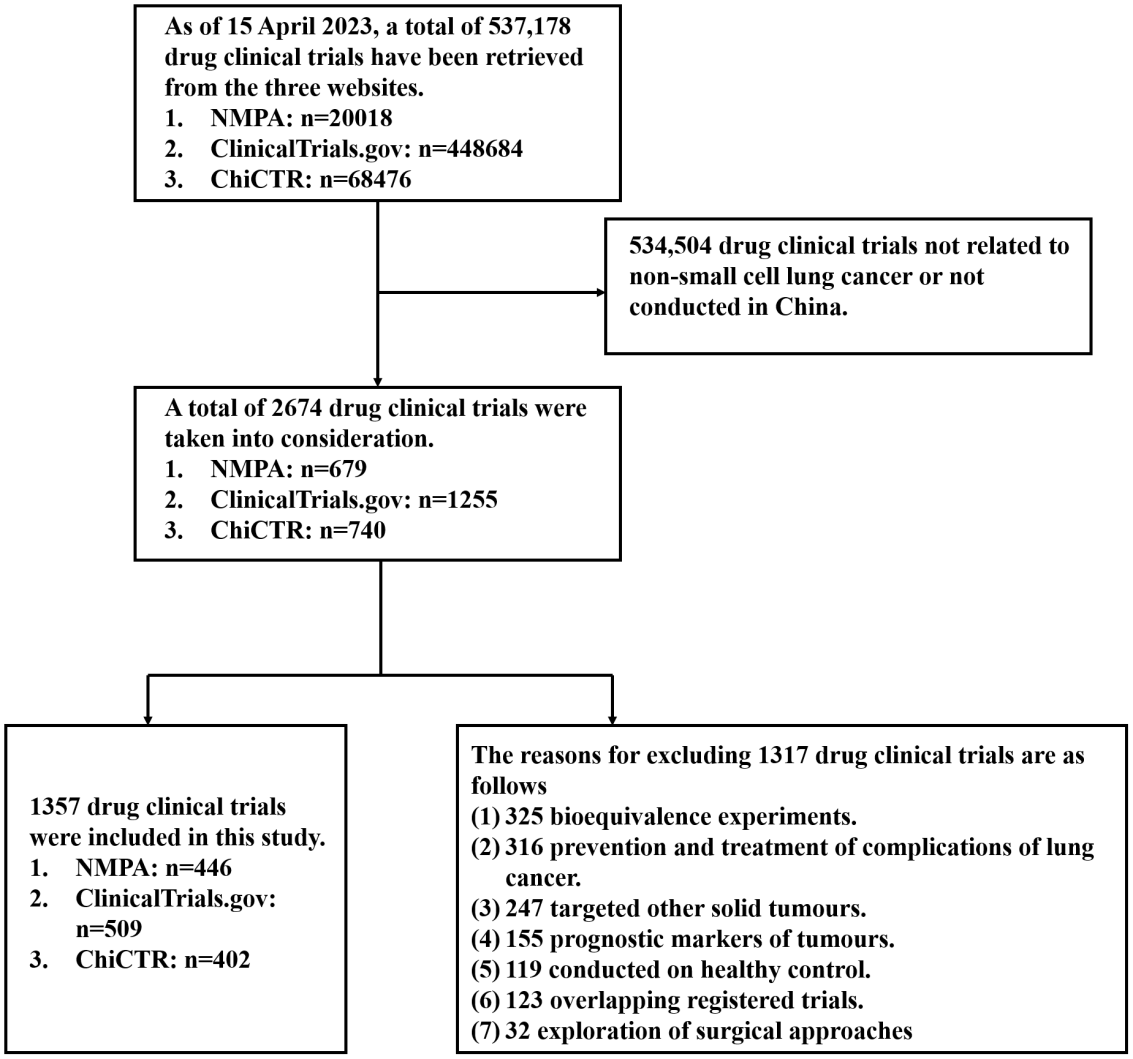
The full screening process for drug clinical trials.

Based on the above inclusion and exclusion criteria, we screened 1,357 clinical trials of drugs relevant to the treatment of NSCLC that were conducted in China. Figure 1 shows the full screening process for the studies.

### 2.3 Data extraction and definition

The following information was collected on the website: registration number of the trial, official name of the trial, year of first publication, type of drug in the trial, trial conduct status, sponsor, address of the lead unit, address of the participating unit, trial phase, type of design, randomization, and whether the protocol was co-administered. Two investigators independently downloaded drug clinical trial data from three websites, which were then identified. All discrepancies were discussed by all investigators, and data were carefully identified and screened.

### 2.4 Statistical analysis

We have analyzed temporal trends in specific indicators, including the number of clinical trials screened at the three sites, the status of clinical trials of drugs at various stages, the geographical distribution of trial centers, the number of clinical trials involving different types of drugs, and the distribution of clinical trials based on various sponsorships. Statistical significance of differences was evaluated using assessed by student’s *t*-test and one-way ANOVA followed by Tukey test (GraphPad Software, San Diego, CA), and *p* < 0.05 was considered statistically significant. We used R software (version 4.2.2) to collate and analyse the data.

## 3 Results

### 3.1 Time trends of clinical trials by study phase

We screened a total of 1,357 drug clinical trials. Of the total drug clinical trials, 157 were observational studies, accounting for 11.57% of the total, while the other 1,200 were interventional studies, accounting for 88.43% of the total.

**Figure 2:**
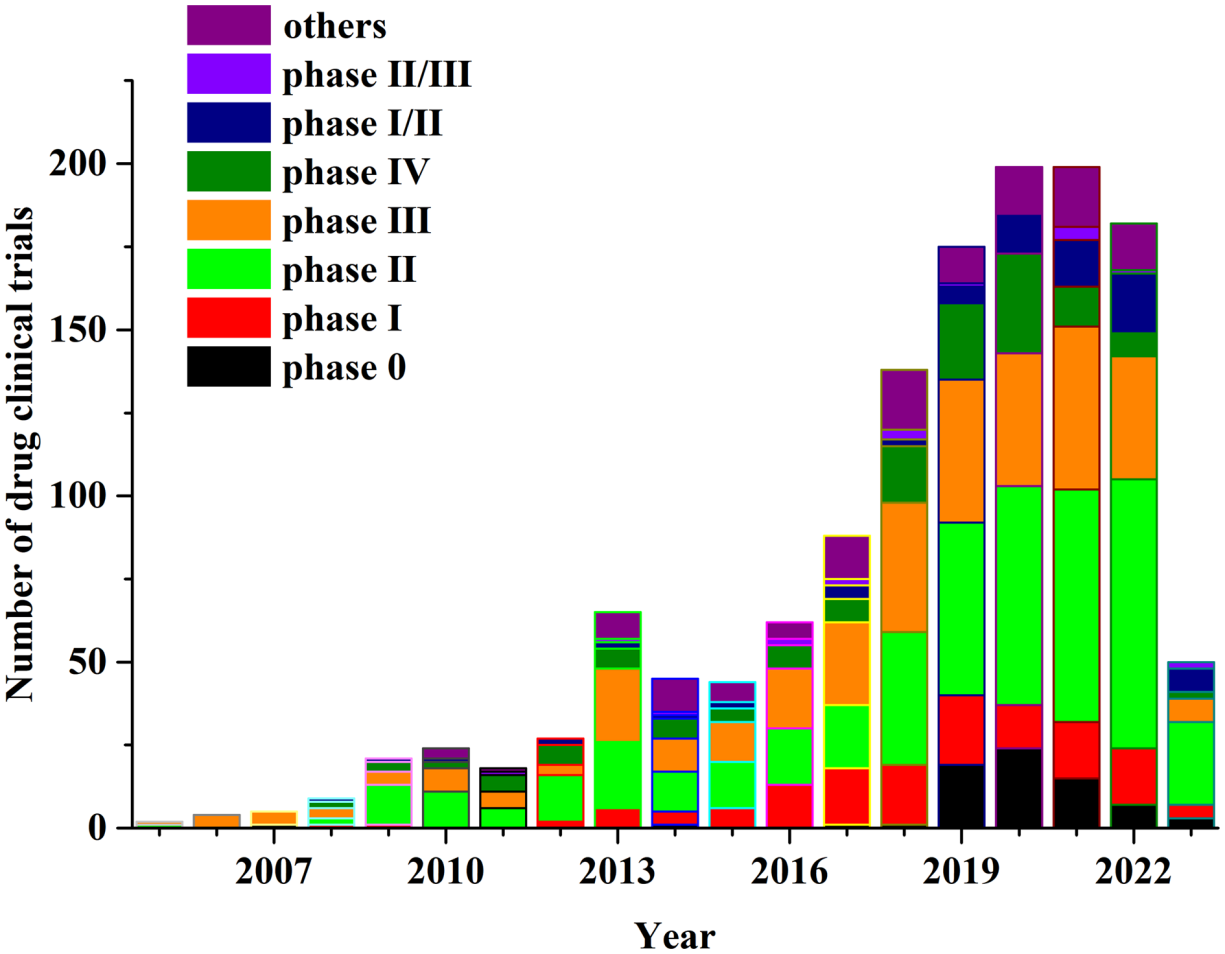
Annual number of clinical trials of NSCLC drugs conducted in China based on study phase from 2005 to 2023.

Of the clinical trials reviewed, 140 were phase I, 462 were phase II, 333 trials were phase III and 139 clinical trials were phase IV, representing 10.32, 34.05, 24.54 and 10.24%, respectively (Figure 2). Of the total drug clinical trials, 72 (5.31%) included Phase I/II and 18 (1.33%) involved Phase II/III. The Food and Drug Administration (FDA) proposed the concept of exploratory investigational new drug (eIND) and in 2006 released the eIND research guide. The studies conducted under an eIND are termed Phase 0 clinical trials (17). Seventy-two (72) Phase 0 drug trials were employed accounting for 5.3% of all drug clinical trials. Overall, 121 (8.92%) observation studies or clinical trials were not staged. From the perspective of research phase distribution, highest proportion were Phase II clinical trials, which as a category had the fastest growth rate.

### 3.2 Distribution and time patterns of clinical trials by sponsorship

Industry sponsored trials (IST) were more prevalent than investigator-initiated trials (IIT). In NMPA, all registered drug clinical trials were ISTs; in the ChiCRT and Clinicaltrial.gov, IIT trial registration also existed. IST drug trials accounted for 53.2% (*n* = 722) while IIT clinical trials were 46.8% (*n* = 635) of the total.

**Figure 3:**
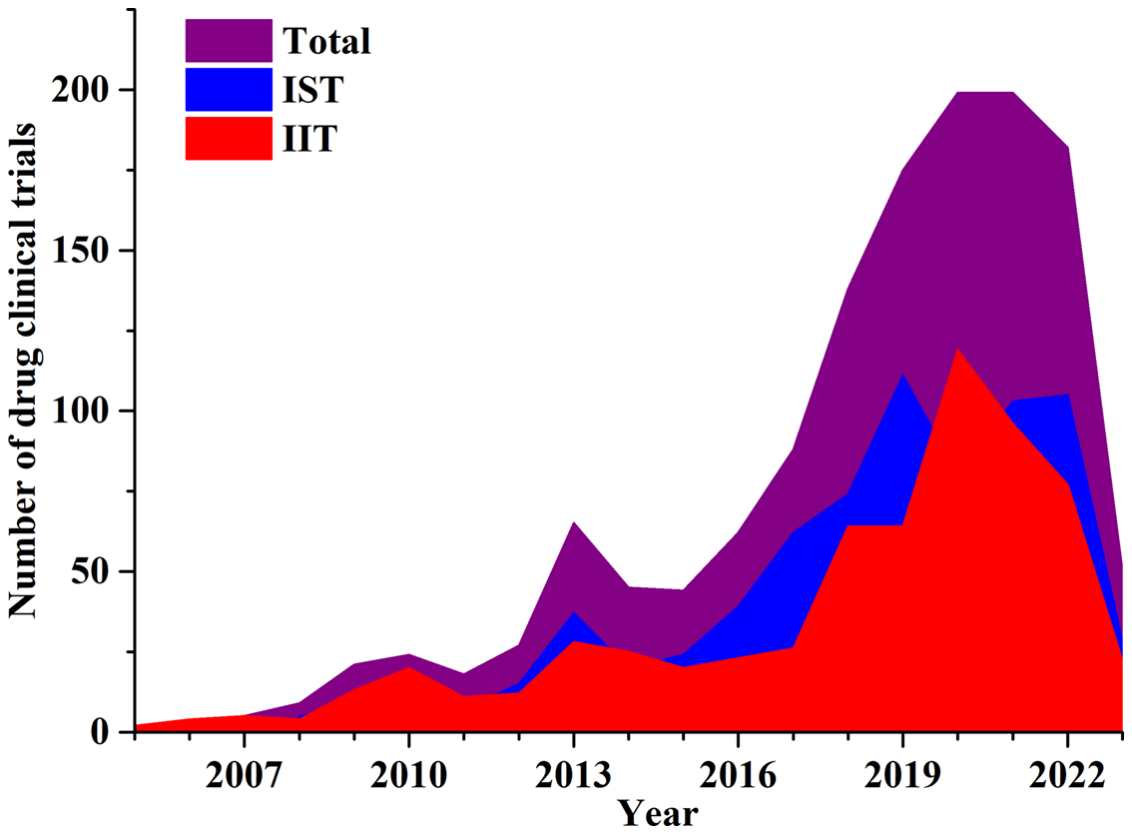
Annual number of clinical trials of NSCLC drugs conducted in China based on sponsorship from 2005 to 2023.

NSCLC related IST clinical trials in China from 2008 to 2022 had an average annual growth rate (AARG) of 24.29%. Similarly, an IIT drug studies showed an upward trend between 2005 to 2020, with the largest annual increase of 146% in 2018 which peaked in 2020 followed by a downward trend thereafter. The AARG of IIT drug trials was 31.31% between 2005 to 2020. The total number of drug candidate studies significantly increased in 2018 and 2019 due to the rapid expansion of IST and IIT clinical trials. The IST research had two small peaks in 2019 and 2021 reflecting a new trend with corresponding growth rates of 50.00% and 28.75%, respectively. Figure 3 illustrates more detailed data on temporal trends in industry clinical trial sponsorship.

### 3.3 Distribution of clinical trials by drug type

**Figure 4:**
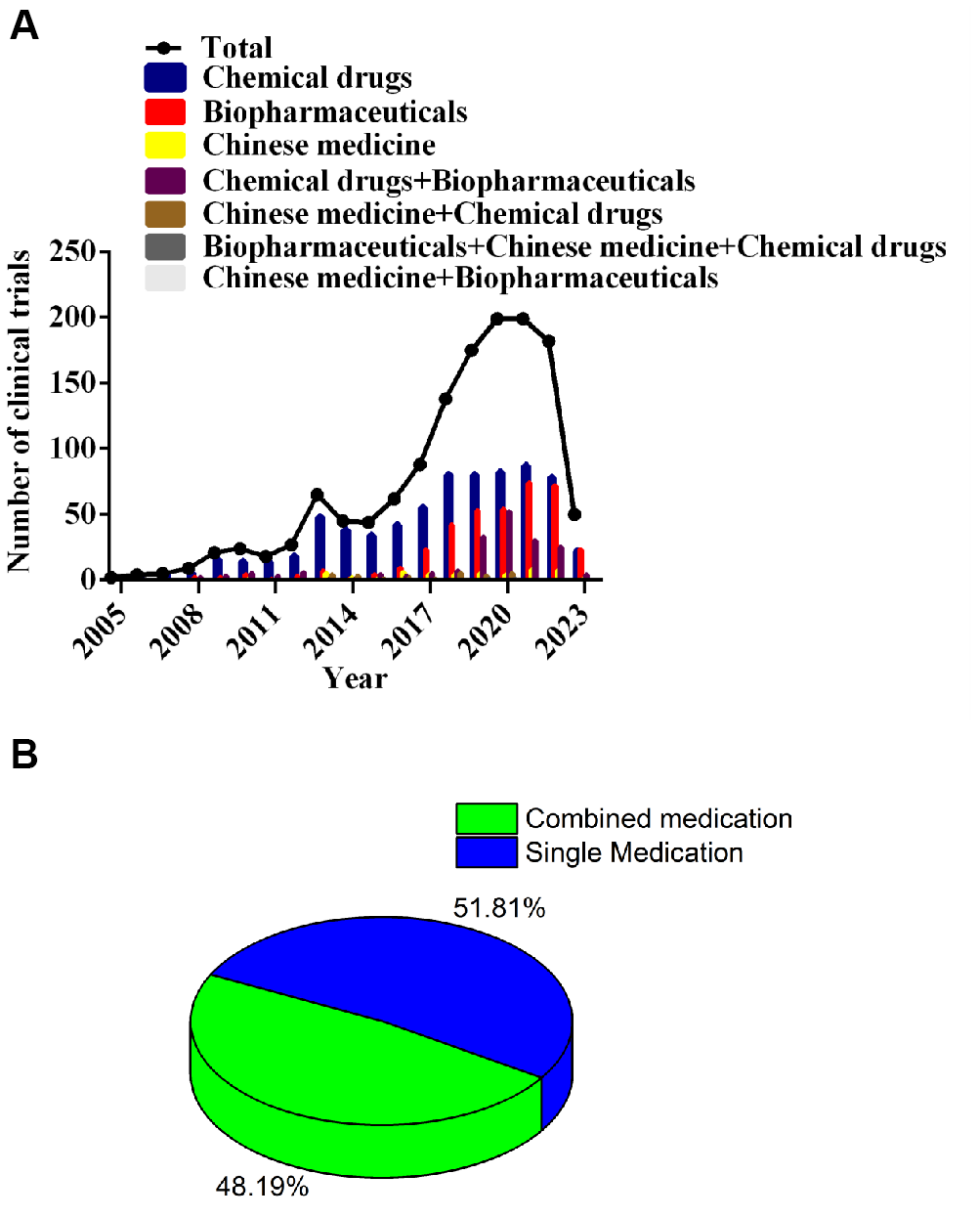
Annual number of clinical trials of NSCLC drugs conducted in China based on drug type from 2005 to 2023 **(A)**; percentage of trials for combined medication versus single medication **(B)**.

Drugs incorporated into clinical trials were classified into seven categories: 53.2% (723/1357) were chemicals, 27.6% (374/1357) drugs were biopharmaceuticals, and 3.5% (48/1357) were Chinese medicines. Chemicals and biopharmaceutical testing had annual increases in clinical trials. The AARG of chemicals was 32.2% from 2005 to 2021 and 32.0% for biopharmaceuticals from 2008 to 2021; both forms peaked in 2021. Some clinical trials for NSCLC used combination drugs (654, 48.2%); whereas, 703 (51.8%) were single drug treatments. Of the combination trials, 13.6% (185/1357) included biopharmaceuticals and chemical drugs, and only 1.8% (24/1357) paired a Chinese medicine with a chemical drug. Figure 4 shows the temporal trends in these trials.

However, we classified the drugs according to the mechanism of action, categorizing the drugs as immune-therapeutics, anti-angiogenics, targeted therapeutics, chemotherapeutics, Traditional Chinese medicines, and others. Among these trials 548 were for targeted therapeutic (40.4%) and 419 were for immunotherapeutic (30.88%). The number of immunotherapeutic drug trials increased annually from 2013 to 2021, reaching a peak of 88 candidates in 2021, which declined slightly to 83 in 2022. Over the 2013 to 2022 interval, AAGR of immunotherapeutic drugs was 63.4%. Targeted therapeutics also rose annually between 2005 and 2022 with an AAGR of 29.3% (Supplementary Figure S1).

### 3.4 Distribution of clinical trials by recruiting status

**Figure 5:**
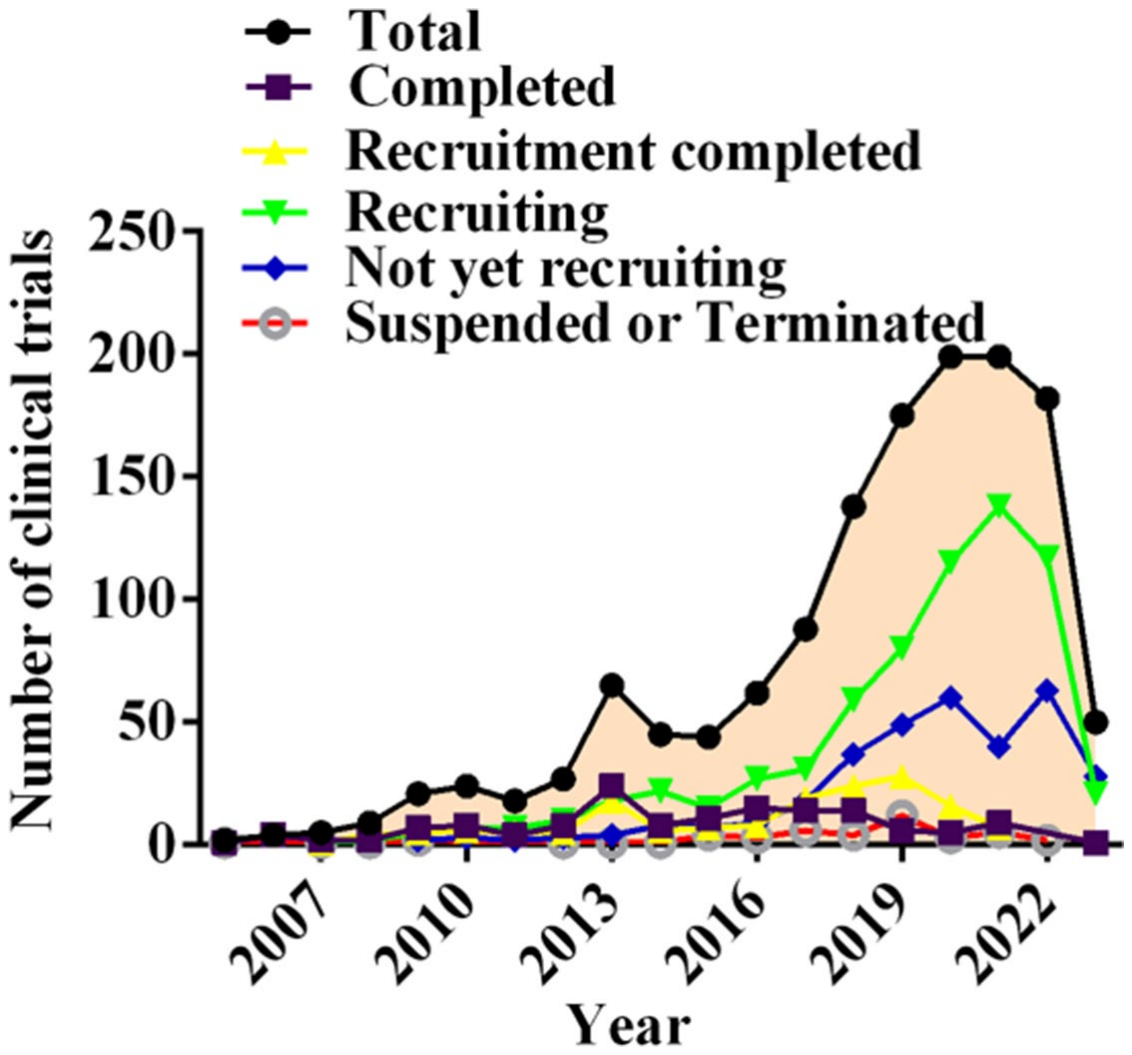
Annual number of clinical trials of NSCLC drugs conducted in China based on recruiting status from 2005 to 2023.

Segmentation of clinical trials according to recruitment status revealed that 143 trials were completed (10.54%), 159 had completed recruitment (11.72%), 675 were still in the recruitment phase (49.74%), and 333 trials had not begun enrolling participants (24.54%). Notably, 47drug candidate trials were suspended or terminated (3.46%). In general, suspended or terminated drug trials were related to changes in the sponsoring company’s strategy or difficulties recruiting suitable patients, perhaps due to stringent inclusion criteria. Interestingly, a large increase in the drug trials enrolled occurred in 2018 (Figure 5).

### 3.5 Time trends of leading clinical trial units

**Figure 6:**
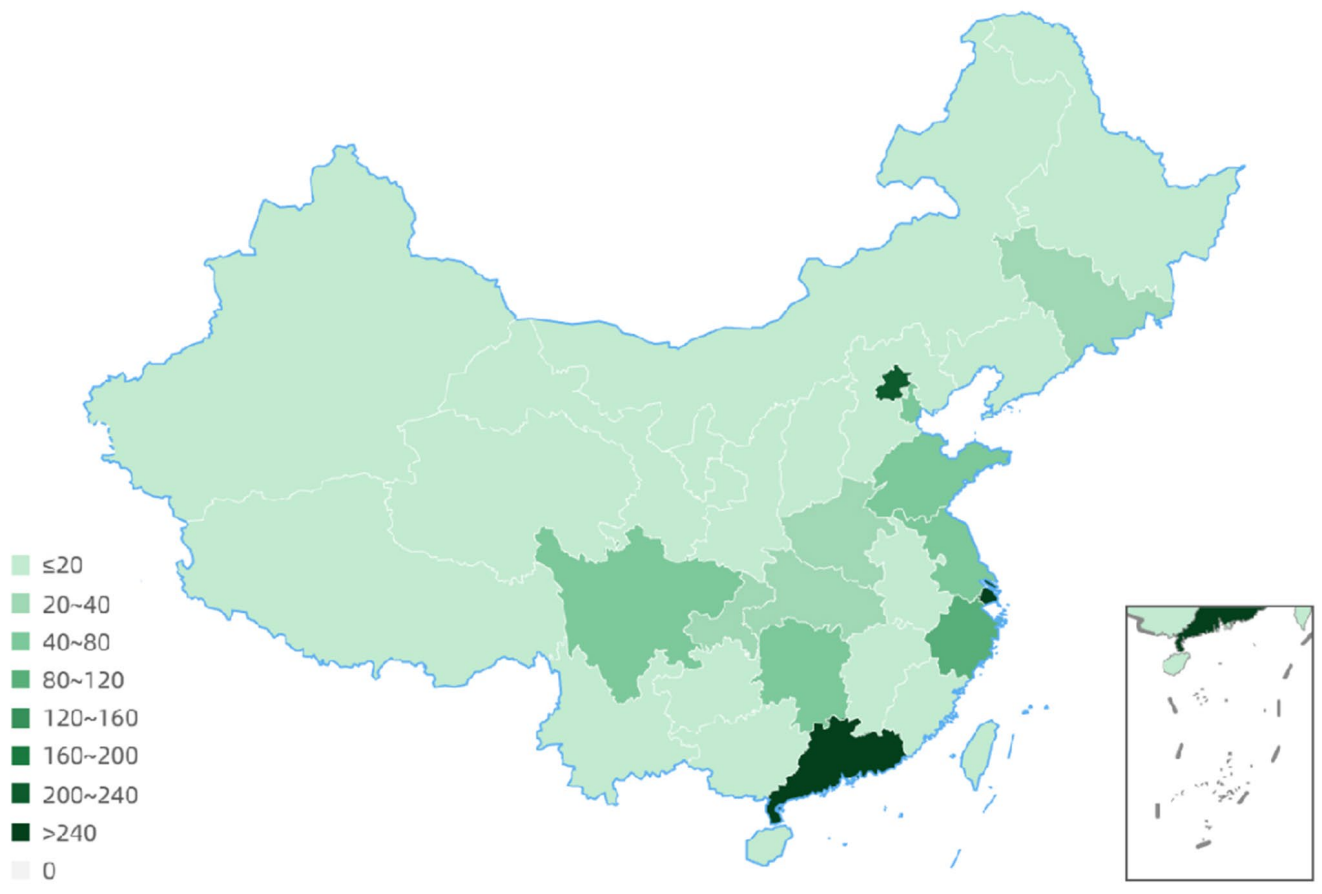
Geographical distribution of drug clinical trial leading units.

The geographical distribution of principle centers for drug clinical trials between 2005 to 2023 revealed that 54 trials had 2 to 7 leadership sites in China and 2 trials had the primary center located abroad. Details for 4 clinical trials were not available. Among all drug trials, 240 (16.7%) lead units were in Beijing; 345 (24.1%) were located in Shanghai, and 267 (18.6%) were in Guangdong province (Figure 6; Table 1).

**Table 1:** Detailed geographical distribution of drug clinical trial leading units.

Region or province of PI unit	Numbers of clinical trials	Region or province of PI unit	Numbers of clinical trials
Beijing	240	Shandong	54
Tianjin	52	Henan	23
Hebei	8	Hubei	27
Shanxi	3	Hunan	41
Inner Mongolia	3	Guangdong	267
Liaoning	5	Guangxi	19
Jilin	37	Hainan	3
Heilongjiang	8	Chongqing	27
Shanghai	345	Sichuan	54
Jiangsu	65	Guizhou	4
Zhejiang	83	Yunnan	3
Anhui	19	Shanxi	15
Fujian	16	Gansu	2
Jiangxi	5	Hong Kong	1
Ningxia	0	Xinjiang	5
Qinghai	0	Tibet	0
Taiwan	0	Macao	0

### 3.6 Geographical distribution of drug clinical trials participating units

**Figure 7:**
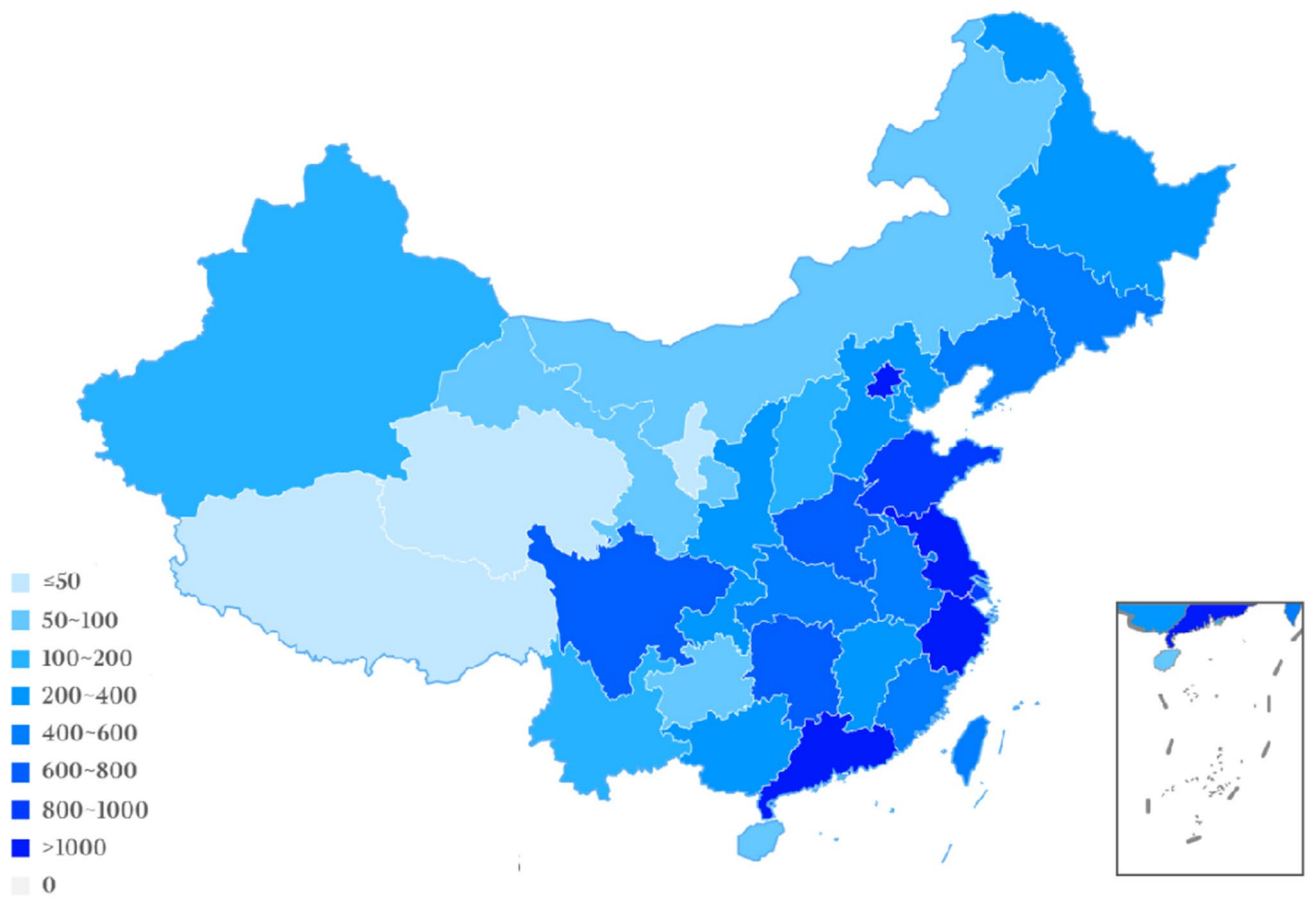
Geographical distribution of drug clinical trial participating units.

Geographic location analysis of the participating trial sites from 2005 to 2023 revealed the top five to be Beijing (*n* = 1,446, 9.8%), Zhejiang (*n* = 1,194, 8.1%), Jiangsu (*n* = 1,063, 7.2%), Guangdong (*n* = 1,046, 7.1%) and Shanghai (*n* = 951, 6.4%). In comparison to these locations, other outlying institution locations participating in drug clinical trials included: Tibet (*n* = 0, 0%), Qinghai (*n* = 8, 0.05%), Ningxia (*n* = 32, 0.2%), and Hainan (*n* = 72, 0.5%). The location information regarding a few drug clinical trials was unavailable. These results demonstrate the maldistribution of NSCLC drug clinical trials towards developed areas, which could present unanticipated patient selection bias. Detailed regional data are presented in Figure 7; Table 2.

**Table 2:** Detailed geographical distribution of drug clinical trial participating units.

Region or province	Numbers to participated in	Region or province	Numbers to participated in
Beijing	1,446	Shandong	922
Tianjin	332	Henan	732
Hebei	318	Hubei	598
Shanxi	158	Hunan	656
Inner Mongolia	90	Guangdong	1,046
Liaoning	441	Guangxi	278
Jilin	429	Hainan	72
Heilongjiang	290	Chongqing	384
Shanghai	951	Sichuan	608
Jiangsu	1,063	Guizhou	76
Zhejiang	1,194	Yunnan	171
Anhui	547	Shanxi	298
Fujian	482	Gansu	76
Jiangxi	286	Qinghai	8
Ningxia	32	Xinjiang	138
Hong Kong	121	Tibet	0
Taiwan	514	Macao	0

### 3.7 Time trends of other characteristics of clinical trials

Additional analysis of the clinical trials site data revealed that 200 (14.7%) were international multi-center clinical trials, 609 (44.9%) were domestic multi-center trials, 137 (10.1%) were domestic single center studies, and the remaining clinical sites were unspecified in the trial registration. Trial experimental trial designs included 621 (45.8%) single-arm, 401 (29.6%) controlled, and 42 (3.1%) cohort studies. Patient randomization was performed in 609 (44.9%) and 748 (55.1%) were nonrandomized trials. Blinding is an important measure to control trial bias, as guided by the CDE Guidelines for Clinical Trials of Drugs. For randomised clinical trials, blinding is usually combined with randomised grouping and works throughout the trial to maximise the control of trial bias. Treatment blinding in this study was categorized as open, double-blind, single-blind. The majority of trials were open (1101), but 220 were double-blind, 3 were single-blind, 18 were retrospective research and 15 trials were prospective studies.

## 4 Discussion

The 2020 global cancer statistics released by the International Agency for Research on Cancer (IARC) (18), reported lung cancer as the second most common malignant tumor type, which also has the highest fatality rate in the world (19, 20). In 2020, there were 820,000 lung cancer cases and 710,000 deaths, accounting for 17.9% of the incidence of cancer in China and 23.8% of the national mortality rate (18). Histological identification confirmed that the most common subtype was NSCLC. Lung cancer develops via the activating mutations of driver genes, which include the epidermal growth factor receptor (EGFR), anaplastic, lymphoma kinase (ALK), c-ros oncogene 1 (ROS1), v-Raf murine sarcoma viral oncogene homolog, B (BRAF), and (RET) genes (21) with rearrangement at some transfection sites. These mutant genes constitute the biological pathways for targeted anti-NSCLC therapy. In the recent past, the treatment of NSCLC focused on surgical resection, radiotherapy, chemotherapy and targeted therapy. Among these, chemotherapy is mainly founded upon on highly toxic platinum drugs. Adjuvant firstline treatment consists of a doublet chemotherapy regimen derived from paclitaxel, gemcitabine, docetaxel, pemetrexed, vinorelbine or etoposide given in combination with either cisplatin or carboplatin (22). The latest Clinical Practice Guideline for Primary Lung Cancer (2022 version) released by the General Office of the National Health and Health Commission of People’s Republic of China (23), classified the NSCLC treatments into four categories: chemotherapy, anti-vascular treatment, immunotherapy and targeted therapy. Anti-vascular drugs were predominantly vascular endothelial inhibitory protein or Bevacizumab. Immunotherapy drugs were PD-1 and PD-L1 inhibitors (Table 3). In recent years, the treatment approaches to NSCLC have evolved in light of the success of immune checkpoint inhibitors (ICIs) and anti-vascular generation drugs often combined with traditional drugs of varying mechanisms.

**Table 3:** NSCLC China guide recommended commonly used drugs (23).

Classification of drugs by mechanism	Drugs
Anti-angiogenic drugs	Vascular endothelial inhibitory protein
	Bevacizumab
Immunotherapeutic drugs	Sintilimab
	Tislelizumab
	Camrelizumab
	Nivolumab
	Pembrolizumab
	Atezolizumab
	Durvalumab
Targeted therapeutic drugs	Gefitinib
	Erlotinib
	Icotinib
	Dacomitinib
	Afatinib
	Osimertinib
	Crizotinib
	Alectinib
	Ceritinib

Numerous reviews of clinical studies have chronicled developments in China and internationally regarding (1) new NSCLC diagnostic and prognostic biomarkers (24, 25); (2) descriptions and analyses of drug trial of combination treatment comparisons (26, 27); (3) recognition susceptible genes in lung cancer (28, 29); (4) changes of RNA and protein in the tissue of NSCLC of patients, and understanding pathways underpinning the development NSCLC (30). Despite these focused papers, no overarching and systematic study regarding development of clinical trials for NSCLC’s treatment has been considered, until this comprehensive evaluation of the numerous trends in clinical trials for NSCLC conducted in China.

Of these, chemical drugs accounted for the largest proportion (53.3%) of NSCLC treatments (Figure 4), possibly reflecting the established chemotherapy approach to cancer. Nonetheless, clinical trials evaluating biopharmaceuticals have gradually increased in the past 10 years, and by 2021, this class of drug treatment represented largest proportion tested (52.3%), reflecting increased popularity of biopharmaceuticals-based treatment approaches for NSCLC over the past 20 years. Considering all clinical trials, 123 clinical programs were bioequivalent research testing generic or analogue versions of approved drugs. Clinical trials for new drug entities were represented by immune-therapeutics (30.9%), anti-angiogenics (15.1%), targeted therapeutics (40.4%), chemotherapeutics (7.2%), a small number of Traditional Chinese medicines (5.5%) and others (1.0%). Targeted therapies account for the greatest proportion of these drugs (Supplementary Figure S1). In 2015, the Chinese government formulated new policies to boost innovative drugs through priority review status (31). In the same timeframe (2017), the Chinese government issued the “Opinions on Encouraging Medical Device Innovation to Reform the Review and Approval System” that encouraged innovative drug development (32). Collectively, these policies promoted continuously increased clinical evaluation of innovative NSCLC drugs, alone or in combination, yielding more novel and updated NSCLC treatment methods.

Despite advances in lung cancer treatment with third-generation chemotherapies and targeted therapies, prognosis for NSCLC patients remained poor. More recently inhibitory immunotherapies targeting cell programming death receptors 1(PD-1) and cell programming death ligand 1(PD-L1) have emerged as the newest treatment for NSCLC (33, 34). These therapeutics generally classified as immune checkpoint inhibitors (ICI) are monoclonal antibodies defined by three subgroups: PD-1 inhibitors (nivolumab, pembrolizumab), PD-L1 inhibitors (durvalumab, atezolizumab, and avelumab), and cytotoxic T-lymphocyte-associated protein 4 (CTLA-4) inhibitors (ipilimumab) (33, 35). The ICI therapy has markedly improved the clinical response and survival rates among patients with metastatic NSCLC. In May 2017, the US FDA approved the combination of nivolumab plus ipilimumab for the treatment of non-squamous NSCLC. On October 12, 2021, China officially listed CTLA-4 inhibitor ipilimumab and the combination of nivolumab plus ipilimumab has been approved as the hypertrophic inhibitor therapy. These events marked China’s domestic era of dual-immune inhibitors treatment era (36). Currently, dual immune checkpoint drugs, PD-(L)1 plus CTLA-4 inhibitors, has become one of the most widely prescribed treatment approaches for metastatic NSLC. Moreover, some studies suggest that neoadjuvant nivolumab in combination with ipilimumab improves the primary pathological response in early-stage NSCLC, regardless of PD-L1 expression, suggesting, that dual-ICI offered long-term survival benefits (37–39). Others suggest that the combination of the dual immune checkpoint agents was not advantageous as a neoadjuvant regimen compared to single agent treatment (40). Of note, during clinical development, some researchers terminated ICI clinical trials (41) due to the significant adverse reactions (ADRs). Immunotherapy receptor targets are distributed into many organ systems and ICIs, particularly dual-inhibitor regimens, induced potentially fatal off-target toxicities including immune-related pneumonia, myocarditis, encephalitis, and nephritis. Part of the ADRs and severity grades are listed in Table 4. These events endorsed treatment recommendation revisions, warnings, or contraindications for patients with underlying immune or autoimmune disease. Consequently, continued clinical study of nivolumab plus ipilimumab among the NSCLC patients continues, with the hope to define predictive or early biomarkers of adverse events.

**Table 4:** Treatment-related adverse events (TRAEs) with possible or probable attribution to study therapies (38, 39, 41–43).

Treatment arm	Events	Grade 1–2	Grade 3–5	Attrition
Ipilimumab + Nivolumab	Febrile neutropenia		✓	Treatment related
Ipilimumab + Nivolumab	Rash	✓	✓	Treatment related
Ipilimumab + Nivolumab	Pruritus	✓	✓	Treatment related
Ipilimumab + Nivolumab	Fatigue	✓		Treatment related
Ipilimumab + Nivolumab	Acute respiratory distress syndrome (ARDS)		✓	Treatment related
Ipilimumab + Nivolumab	Headache		✓	Treatment related
Ipilimumab + Nivolumab	Pneumonitis		✓	Treatment related
Ipilimumab + Nivolumab	Abdominal pain	✓		Treatment related
Ipilimumab + Nivolumab	Arthralgia	✓		Treatment related
Ipilimumab+ Nivolumab	Diarrhea/colitis	✓	✓	Treatment related
Ipilimumab + Nivolumab	Fever	✓		Treatment related
Ipilimumab + Nivolumab	Hypothyroidism	✓		Treatment related
Ipilimumab + Nivolumab	Infusion reaction	✓		Treatment related
Ipilimumab + Nivolumab	Nausea	✓		Treatment related
Ipilimumab + Nivolumab	Psoriasis	✓		Treatment related
Ipilimumab + Nivolumab	Decreased appetite	✓	✓	Treatment related
Ipilimumab + Nivolumab	Vomiting	✓	✓	Treatment related
Ipilimumab + Nivolumab	Neutropenia	✓		Treatment related
Ipilimumab + Nivolumab	Anemia	✓	✓	Treatment related

Rather than administering preformed antibodies, new clinical studies are probing the efficacy and safety of antibody-generating drugs, spawned from the evolving identification of targetable oncogenic driver alterations (44). An expanding category of oral targeted therapy options is under study for first-line use in treatment-naïve setting (45). Although oral targeting drugs have a high benefit rate in advanced NSCLC, the total survival period and the development drug resistance continues. Anti-vascular drugs reduce immunosuppression by modulating the tumor micro-environment (TME). This therapy is particularly effective when combined with other treatment NSCLC drugs (small molecular targeted therapy, chemotherapy, immunotherapy, etc.), potentially providing anti-tumor, anti-drug resistance benefits, and diminished ADR benefits (46–48). In recent decades, more than 10 anti-angiogenic therapeutics including bevacizumab, regorafenib and sorafenib have been approved for the therapy against several malignant diseases (49–51). A large number of drug clinical trials now include anti-vascular endothelial growth factor (VEGF) monoclonal antibodies such as bevacizumab, in combination drug regimens. Moreover, an increasing pre-clinical literature, suggests that targeting of the VEGF axis may not only inhibit angiogenesis, but may also trigger anti-cancer immunity (52).

Clinical trials excluded in this review studied the changes of ctDNA to track the progression of early-stage lung cancer, which may be extended to interrogate of oncogenic drivers and track expression of resistant mutations. Future clinical trials based on ctDNA-therapeutics are anticipated (53). By evaluating the dynamic changes of ctDNA values in different parts of the tumor cells may refine diagnoses and drug treatment regimens. The thrust of new clinical trials may be to individualize treatments for specific gene mutation groups. Continued exploration of NSCLC biomarkers and immune checkpoint inhibitor biology may evolve into precision medicine guidance and selection of therapeutic targets, like tyrosine kinase inhibitors (54).

Since 2015, the number of clinical trials for the treatment of NSCLC has increased rapidly, and substantial progress has been achieved. But problems remain to be resolved. First, many clinical trials are evaluating the drug or a biological equivalent. Certainly, exceptional drug development costs and the goal to optimize the use of each entity is compelling. But the shortcomings discovered in current drugs represent unmet need opportunities for new mechanistic approaches. Secondly, the drug clinical trial sites are overly concentrated in economically and academically developed centers: Beijing, Shanghai, Jiangsu, Zhejiang; however, this may induce a populational diversity bias (Figures 6, 7). Regions such as Qinghai, Gansu, and Ningxia have low participation in drug trials. Given the ethnic heterogeneity of Chinese patients nationally, the regional distribution studied is very uneven (Tables 1, 2). Thirdly, China vigorously promotes the application of traditional Chinese medicine. Yet, in the drug clinical trials registered, only 48 (3.5%) studied Chinese medicines. Although the mechanism of action of many traditional Chinese medicine products is still unclear, the therapeutic effects based on extensive experience is established. Traditional Chinese medicine has been shown to modulate tumor proliferation, cell adhesion, apoptosis, and tumor migration, impact angiogenesis and alter immune responsiveness (55–57). Mechanistic understanding underpinning Traditional Chinese medicine treatments in NSCLC need to be mined for new unanticipated opportunities. Our research provides a clear direction for the conduct of clinical trials and lays some groundwork for the development of NSCLC drugs.

This review has limitations. First, registration information provided by different official websites are inconsistent. For instance, the clinical trials of many domestic multi-centered clinical trials in ClinicalTrials.gov do not specifically identify the supporting clinical centers. Second, there is no general uniformity among the inclusion criteria and exclusion criteria in different trials, leading to interpretive challenges comparing or combining study results. Third, the data in this review were collected from 2005 onward, since prior to 2005 registration of clinical studies was not required. Fourth, searches using non -small cell lung cancer as keywords may have passed over studies unwittingly. Finally, when the registry website was first set up, there was insufficient awareness of the research information register among researchers and sponsors, and there was a discrepancy between the early data and the actual conduct.

## 5 Conclusion

In short, this review systematically analyzed the clinical trials and time trends registered for the treatment of NSCLC from 2005 to 2023. In the 18 years reviewed, a rapid rise in the development of biopharmaceuticals, particularly for immunotherapy, have been the most prevalent and effective, improving quality of life and survival. At the same time, our summary of ADRs to the therapy of NSCLC’s dual-immune inhibitors also further revealed the application of CTLA-4 plus PD- (L)1 in other related cancers. However, the research and development of new drug classes and mechanisms is limited. China, and the world broadly, should continue to invest time and resources to address this drug diversity shortcoming.

## Data availability statement

The datasets presented in this study can be found in online repositories. The names of the repository/repositories and accession number(s) can be found in the article/Supplementary material.

## Author contributions

WJ, HY, LS, JW, SN, GZ, ML, JL, and RN contributed to the concept of the research question and study design. HY, SN, and WJ searched databases, analyzed data, and prepared the manuscript. JL and RN revised the article. LS and ML designed the study. ML and JW prepared figures and/or tables. GZ and ML did manuscript editing. All authors contributed to the article and approved the submitted version.

## Footnotes

^1^ http://www.cde.org.cn/

^2^ https://clinicaltrials.gov/

^3^ http://www.chictr.org.cn
