# Supplementary material for: Development of clinical trials for non-small cell lung cancer drugs in China from 2005 to 2023
Source: Front Med (Lausanne). 2023 Nov 16;10:1239351. doi: 10.3389/fmed.2023.1239351 (PMC10687557; doi:10.3389/fmed.2023.1239351)
Supplement: Supplementary file 1 [file Data_Sheet_1.DOCX]

Supplementary Material

**Development of clinical trials for non-small cell lung cancer drugs in China from 2005 to 2023**

Wanying Jia^1^, Haiyan Yu^1^, Li Song^2^, Jian Wang^3^, Shuyu Niu^1^, Guojie Zang^4^, Mingjie Liang^1^, Jinwei Liu^1*^, Risu Na^5*^

*** Correspondence:** Jinwei Liu and Risu Na: L18047664042@163.com and nrsmail@163.com

# Supplementary Figures and Tables

## Supplementary Figures


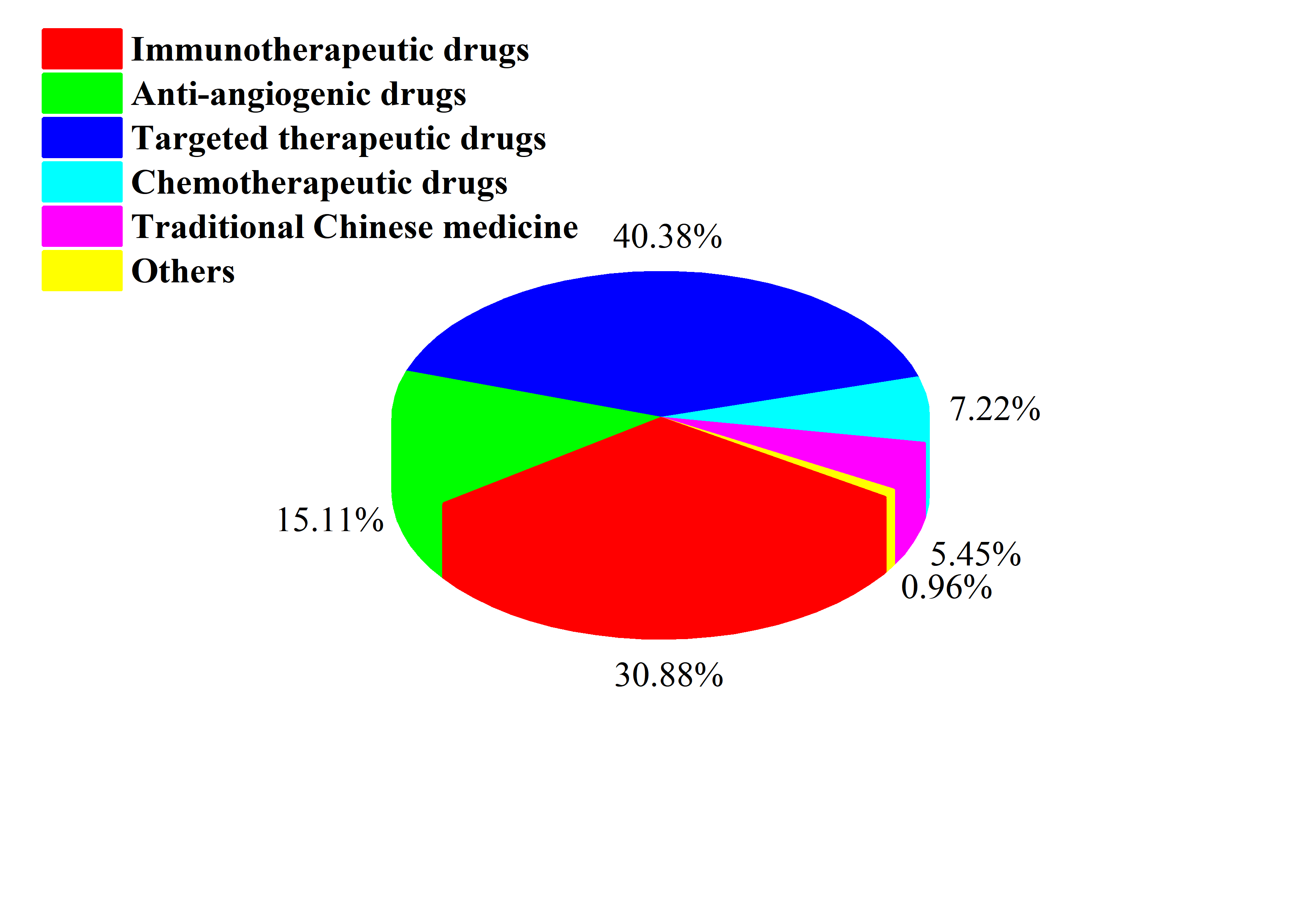


**Supplementary Figure S1.** Annual number of clinical trials of NSCLC drugs conducted in mainland China based on the mechanism of drug action from 2005 to 2023
